# Endogenous expression of UNC-59/Septin in *C.* *elegans*

**DOI:** 10.17912/micropub.biology.000200

**Published:** 2019-12-20

**Authors:** David D. Chen, Eric Hastie, David R. Sherwood

**Affiliations:** 1 Duke University, Department of Biology; 2 Duke University, Department of Biology, Regeneration Next

**Figure 1:**
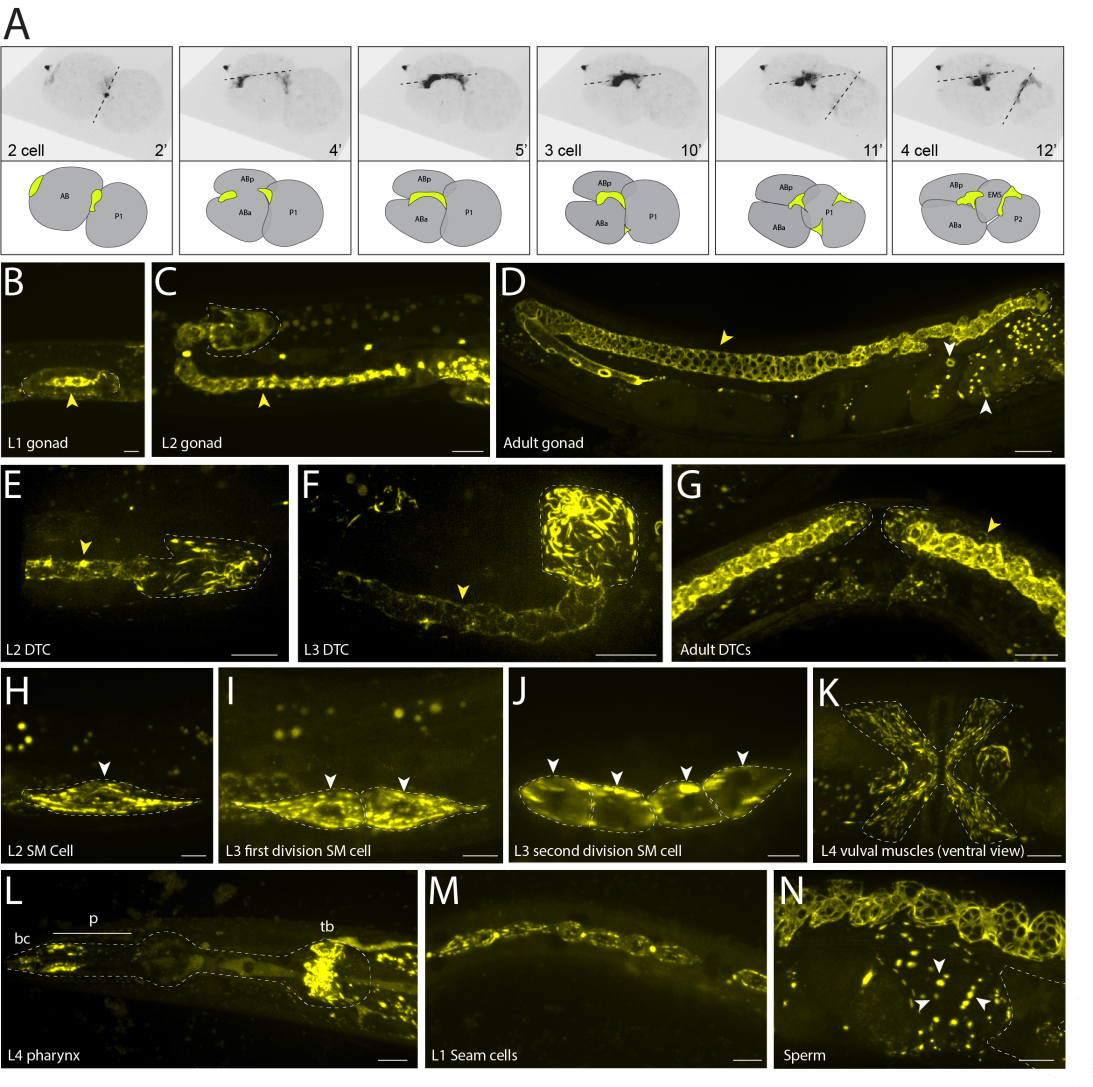
Max intensity projections showing live imaging of GFP fluorescence and localization of endogenously tagged UNC-59::GFP::3xflag::AID/Septin during *C. elegans* development. A. Embryonic cells divisions. Embryo divisions from the 2- to 4-cell stage (time in minutes, black dashed lines depict division planes). Illustration shows dividing AB and P1 cells and UNC-59::GFP localization at cleavage furrows (yellow) B-D. Gonad morphogenesis. The distal tip cells (DTC) (white dashed line), which leads gonad outgrowth, and the rachis (yellow arrowheads) that supports the developing germline syncytium. In D, white arrowheads mark embryonic cell cleavage rings. E-G. DTC migration. The DTC throughout its L2 to young adult stage migration with septin bundles and rings present (white dashed line, yellow arrowhead indicates rachis where septin form rings in germ cells to maintain a syncytium). H-K. Sex myoblast divisions. A migrating sex myoblast (white dashed line) during the L2 stage. After migration, the sex myoblast and its descendants continue to express UNC-59 during divisions in the L3 stage once these cells form the vulval muscles (white dashed line) in the late L4 stage (ventral view, K). L-M. Pharynx, seam cells and sperm. UNC-59/Septin localization in cells of the pharynx (white dashed line, buccal cavity (bc), anterior of the procorpus (p), and terminal bulb (tb)) in epithelial seam cells, and sperm (white arrows) that have been expelled from the spermatheca (white dashed line) by an embryo during ovulation. All scale bars are 10um.

## Description

Septins are cytoskeletal proteins involved in cytokinesis, morphogenesis, and cell migration. Misregulation of septin expression occurs in neurodegenerative diseases and cancers (Angelis and Spiliotis 2016). Originally discovered in yeast, there are 13 mammalian septins and two in *C. elegans*, *unc-59* and *unc-61* (Hartwell 1971, Nguyen et al. 2000). Here, with live imaging of green fluorescent protein (GFP) fluorescence, we characterize UNC-59/Septin expression and localization throughout *C. elegans* development. Using CRISPR/Cas-9, we endogenously tagged *C. elegans* *unc-59*, which is most similar to human septins 1,2,4,5, and 7 (Kim et al. 2018), with GFP for visualization and live imaging using high resolution confocal microscopy. We first show the localization of UNC-59 at the cleavage furrow (previously shown with antibody staining, (Nguyen et al. 2000)) during a time lapse of cell divisions in early 2- to 4-cell stages of embryogenesis (Fig. 1A) and throughout embryogenesis (cleavage rings in older embryos in Fig. 1D). Septins are also important for gonad morphogenesis and distal tip cell (DTC) migration (Nguyen et al. 2000) where UNC-59 protein is detected throughout gonad development in the rachis (previously shown with endogenously tagged unc-59::mKate, (Priti et al. 2018)) and DTCs (Fig. 1B-G). We highlight UNC-59/Septin localization in the DTC (previously shown with a transgene, (Finger et al. 2003)) at the L2 and L3 stages where it is organized into bundles (DeMay et al. 2011) and ring structures (Figs. 1E and F). The two bilateral sex myoblast cells express UNC-59 during their posterior to anterior migration in the L2 and early L3 stage (Fig. 1H) and continue to express UNC-59 in these cells as they differentiate into vulval muscles in the late L3 to early L4 stages (Fig. 1K). Lastly, we show UNC-59/Septin expression and localization in tissue not previously reported: in the pharynx (cells of the buccal cavity, anterior procorpus, and terminal bulb) (Fig. 1L); in the seam cells, both in bundles and at the cleavage furrows, beginning in the L1 stage (Fig. 1M) and continuing throughout development and into the adult; and in sperm surrounding an embryo that has exited the spermatheca (Fig. 1N).

## Reagents

CRISPR constructs were generated using a self-excising cassette (SEC) for drug (hygromycin) selection as described previously (Dickinson et al. 2015). Guide plasmids were generated using plasmid pDD122 (Peft-3::Cas9 + ttTi5605 sgRNA); Addgene 47550. SG: AAGAAACGAATGGGCGGTCTCGG. To generate *unc-59(qy88[unc-59::GFP::3xflag::AID+loxP])*, pDD282 (GFP-C1^SEC^3xFlag^AID) was modified (Addgene 66823). Primers to generate the homology arms (amplified from gDNA): 5’ F: tacgactcactatagggcgaattgggtaccacaactagtCGTAATGTTCATTATGAGAAT

5’ R: TGGGACAACTCCAGTGAACAATTCTTCTCCTTTACTCATGTTTCGATTAAACAATCCtAatCCtCCCATTCGTTTCTT

3’ F: AAATCAAGCGGTGGCCCGGAGGCGGCGGCGTTCGTGAATAATTCCCTCATTTTTTAAACG

3’ R: agggaacaaaagctggagctccagcggccgctttgcatgCATGTCTTTGTAATGCTGTGG

The first two codons of GFP were removed to generate a direct fusion after a recommendation from Amy Gladfelter at UNC Chapel Hill.
